# Real-Time State Estimation in a Flight Simulator Using fNIRS

**DOI:** 10.1371/journal.pone.0121279

**Published:** 2015-03-27

**Authors:** Thibault Gateau, Gautier Durantin, Francois Lancelot, Sebastien Scannella, Frederic Dehais

**Affiliations:** ISAE (Institut supérieur de l’aéronautique et de l’espace), Toulouse, France; University of California, San Francisco, UNITED STATES

## Abstract

Working memory is a key executive function for flying an aircraft. This function is particularly critical when pilots have to recall series of air traffic control instructions. However, working memory limitations may jeopardize flight safety. Since the functional near-infrared spectroscopy (fNIRS) method seems promising for assessing working memory load, our objective is to implement an on-line fNIRS-based inference system that integrates two complementary estimators. The first estimator is a real-time state estimation MACD-based algorithm dedicated to identifying the pilot’s instantaneous mental state (*not-on-task* vs. *on-task*). It does not require a calibration process to perform its estimation. The second estimator is an on-line SVM-based classifier that is able to discriminate task difficulty (*low working memory load* vs. *high working memory load*). These two estimators were tested with 19 pilots who were placed in a realistic flight simulator and were asked to recall air traffic control instructions. We found that the estimated pilot’s mental state matched significantly better than chance with the pilot’s real state (62% global accuracy, 58% specificity, and 72% sensitivity). The second estimator, dedicated to assessing single trial working memory loads, led to 80% classification accuracy, 72% specificity, and 89% sensitivity. These two estimators establish reusable blocks for further fNIRS-based passive brain computer interface development.

## Introduction

Piloting is a complex activity that takes place in a rapidly changing and uncertain environment. Working memory (WM) is a key executive function for handling the flight, maintaining an up-to-date situation awareness, adapting the flight plan [1, 2] and interacting with ground control [3]. This latter activity, following air traffic control (ATC) instructions, is known to particularly solicit WM as it requires memorization of critical flight information (e.g. heading, altitude, speed) and the input of these parameters into the flight deck. However, human WM is fundamentally limited [4, 5]. Many studies have revealed that several factors such as message length [6, 7] and complexity [8, 9] affect the pilot’s memory capacity necessary for following ATC instructions, as well as their ability to execute commands. One has to consider that the erroneous execution of the ATC clearances may considerably jeopardize flight safety [10], thus prompting the need for enhanced pilot-system interaction.

A promising way to mitigate these human limitations is to consider the implementation of an adaptive system such as a “passive” brain-computer interface (BCI) [11]. “Passive” BCIs are not meant to directly control a device (e.g. a mouse) via brain activity but to support “implicit interaction”. Research on “passive” BCIs provides interesting insight as they aim to infer the human operator’s cognitive state and then adapt the nature of the interactions to overcome cognitive bottlenecks [12, 13] such as WM limitations.

Defining a brain imaging technique to estimate WM load level in an operational context could be considered as the first step required for the development of such an inference system. Functional near-infrared spectroscopy (fNIRS) is an optical brain imaging method that measures cortical hemodynamic response. fNIRS provides good spatial localization compared to EEG, on the order of 1cm^2^, and can be easily integrated with EEG/ERPs [14–16]. Moreover, fNIRS has shown to be correlated with functional magnetic resonance imaging (fMRI) studies [17]. This device is suited for both laboratory and field experiments in flight simulators [18, 19] and has been successfully used to detect WM solicitation [20–24]. Despite these numerous advantages, this technique has been less explored than EEG by the BCI community [11] mainly because slow hemodynamic response prevents real-time interaction with an apparatus [25]. However, fNIRS has been proven to be a suitable technique for BCI purposes [26–30] although most of the demonstrations were achieved in an off-line manner (e.g. [31–34]; for a literature review see [35, 36]). Some on-line BCI have been implemented [25, 37–43] and the processing of fNIRS data in real-time to provide good classification accuracy remains a challenge (e.g. [35, 44]).

Indeed, the on-line extraction of relevant features from the raw fNIRS signal is still a critical issue [45] and new techniques need to be developed to reduce noise and to improve the usability of the data [46, 47]. Several studies [48, 49] have revealed that the Moving Average Convergence Divergence (MACD) filter is a promising processing technique. Relying on the principle of the Exponential Moving Average (EMA) filter, the MACD filter performs efficient fNIRS signal detrending and eliminates the low-frequency drifts and high-frequency physiological and measurement noise from the raw fNIRS signal. It also compares favorably to classical filtering techniques, especially in terms of filter order, and it allows stimulus onset detection without requiring the use of machine learning techniques [50].

Another issue of deriving cognitive activity from the fNIRS signal is related to the selection of the most appropriate features used to discriminate different individuals’ states. Many metrics have been proposed, such as the change in oxyhemoglobin or deoxyhemoglobin concentrations, the difference/sum/ratio of oxyhemoglobin and deoxyhemoglobin amplitudes, time-to-peak, and so forth, but there is still a lack of consensus (for a review of metrics see [51]). In any case, accounting for inter-individual variability is challenging as long as hemodynamics latency and recording sites may differ across participants [52, 53]. To that end, Tai and Chau [54] have proposed to adopt a machine learning approach that considers both spatial (*i.e.* recording sites) and temporal features (*i.e.* time windows). In their paper, the authors processed 208 candidate features such as the mean, kurtosis, skewness, variance, zero crossings, percentages of total energy, and root mean squared of the oxygenated and deoxygenated hemoglobin concentrations for each site through different time windows. This approach provide excellent accuracy off-line, as well as a method for decoding single on-line trials [43].

The aim of this study was to design an on-line fNIRS-based inference system dedicated to:

1. estimating the pilot’s state (performing or not performing a WM task);
2. assessing the WM load level.

To this end, we designed a simplified but plausible pilot-ATC interaction task, using prerecorded messages. The implementation of this inference system was challenging as our participants were placed in an immersive flight simulator (*i.e.* user interfaces, panoramic external “view”) that induces additional cognitive activity (*i.e.* flight trajectory monitoring), as well as motion artifacts (*i.e.* programming autopilot device) therefore adding noise to the fNIRS signal.

To meet these goals, we measured changes in the oxygenation of the prefrontal cortex including the dorsolateral prefrontal cortex (DLPFC) which is known to be involved in WM [18, 22]. Real-time pilot’s state estimation was performed using MACD as proposed in economic market analysis [55] and did not require machine learning techniques. We implemented an on-line single trial classifier [43, 54] to discriminate low WM load achieved trials versus high WM load achieved trials.

## Methods

### 1 Participants

Nineteen visual flight rules (VFR) pilots (6 women; mean group age: 27.4 ± 6.4; mean flight hours 145 ±45) completed the experiment. Pilots had normal or corrected-to-normal vision, normal hearing, and no psychiatric disorders. They all had medical clearance to fly. After providing written informed consent, they were instructed to complete task training. Typical total duration of a subject’s session (informed consent approval, practice task, and real task) was about two hours. This work was approved by the Inserm Committee of Ethics Evaluation (Comité d’Evaluation Ethique de l’Inserm—CEEI/IRB00003888).

### 2 Equipment

#### 2.1 fNIRS Equipment

**Fig 1:**
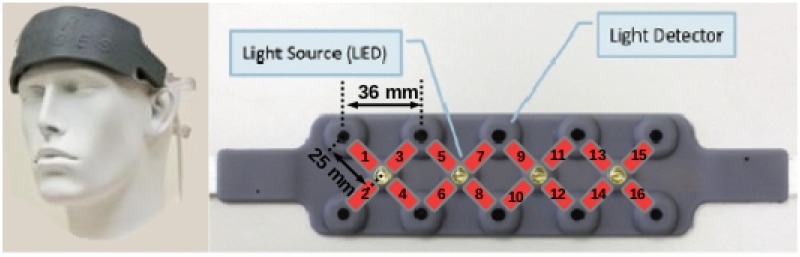
fNIR100® headband and associated channels numbering. Only the four closest detectors to an emitter constituted channels. The emitter-detector distance is 25 mm. Channels are represented in red with their associated number. The original image comes from the fNIRSOFT® manual and has been slightlty modified.

During each experiment, we recorded hemodynamics of the prefrontal cortex using the functional near-infrared spectrometer fNIR100 (Biopac®) equipped with 16 channels (Fig. 1). On this continuous-wave system, the optode separation was about 25 mm and two wavelengths were used, 730 nm and 850 nm.

Each channel of the device records hemodynamics at a frequency of 2Hz in term of oxygenation level variations in comparison to a baseline. Changes in the concentrations of oxygenated (Δ[*HbO*_2_]) and deoxygenated hemoglobin (Δ[*hHb*]) can be calculated from changes in detected light intensity using the modified Beer-Lambert Law [56].

Cognitive Optical Brain Imaging (COBI) Studio® software [57, 58] was used to collect data. The data stream was available on-line from a TCP/IP interface. Before recording, signals for each channel was carefully checked for saturation with COBI Studio which provides signal quality visual representation.

COBI studio was also used to check signal quality and to adjust consequently the headband on the participant’s forehead. Channels 8 and 10, located above the nasal sinus were systematically removed because of saturation [59].

#### 2.2 Flight Simulator

**Fig 2:**
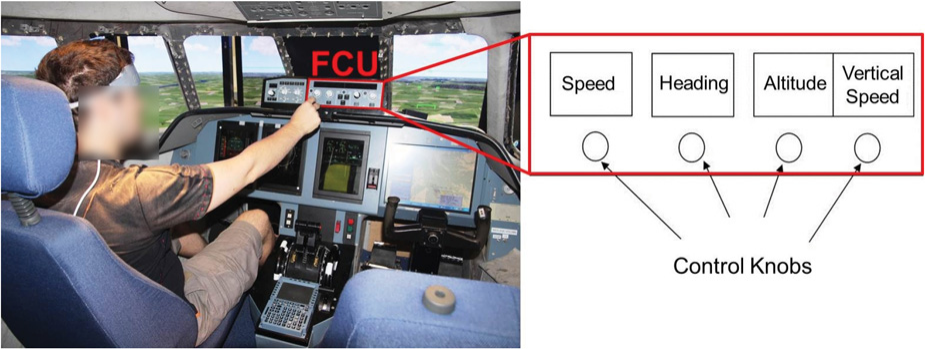
Pilot’s interaction with the FCU. The participants controlled the flight simulator from the pilot’s seat. The red rectangle corresponds to the FCU used to set the autopilot with four control knobs, according to ATC clearances (speed, heading, altitude, and vertical speed selection).

We used the ISAE (Institut Supérieur de l’Aéronautique et de l’Espace—French Aeronautical University in Toulouse, France) flight simulator to conduct the experiment in an ecological situation. It simulates a twin-engine aircraft flight model and the user interface is composed of a Primary Flight Display (PFD), a Navigation Display, and an upper Electronic Central Aircraft Monitoring Display page. The pilot has a Flight Control Unit (FCU) to interact with the autopilot (Fig. 2).

### 3 Protocol

#### 3.1 Task Description

Similar to true flying circumstances, pilots heard ATC messages (pre-recorded for this experiment) and were asked to dial the corresponding flight parameters in the autoflight system using the four knobs (*i.e.* speed, heading, altitude, and vertical speed) of the FCU. The ATC messages were delivered at 78 dB through a Sennheiser® headset. Two levels of difficulty were defined based on the flight parameters that the participant had to set during the experiment:

- *Low WM load*: only one major digit per trial was used to set each flight parameter (e.g: 15 for “speed 150, heading 150, altitude 1500, vertical speed +1500”).
- *High WM load*: each flight parameter value was different from the previous one and composed of different digits to increase the complexity (e.g: “speed 164, heading 235, altitude 8700, vertical speed -1600”).

The task consisted of 20 repetitions of each difficulty for a total of 40 trials. The task difficulty order was randomly distributed with two constraints:

- the first 20 trials contained 10 trials of high difficulty, and 10 trials of low difficulty (which is necessary for machine learning purposes, see 3.3);
- the difficulty cannot be the same for more than two successive trials.

**Fig 3:**
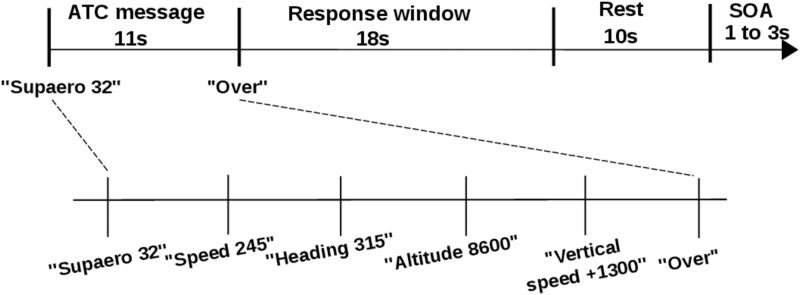
ATC span task trial design.

Each ATC message started with the airplane call sign (i.e. “Supaero 32”), immediately followed by a sequence of flight parameters and ended with the message “over”. Pilots were instructed to set the parameters only after they heard the “over” message (Fig. 3). Thereafter, pilots had to dial the parameters on the autopilot interface during a 18 *s* response window. A practice session was conducted prior to the experiment runs to familiarize them with the experiment protocol and the interface.

#### 3.2 Experimental Components’ Architecture

**Fig 4:**
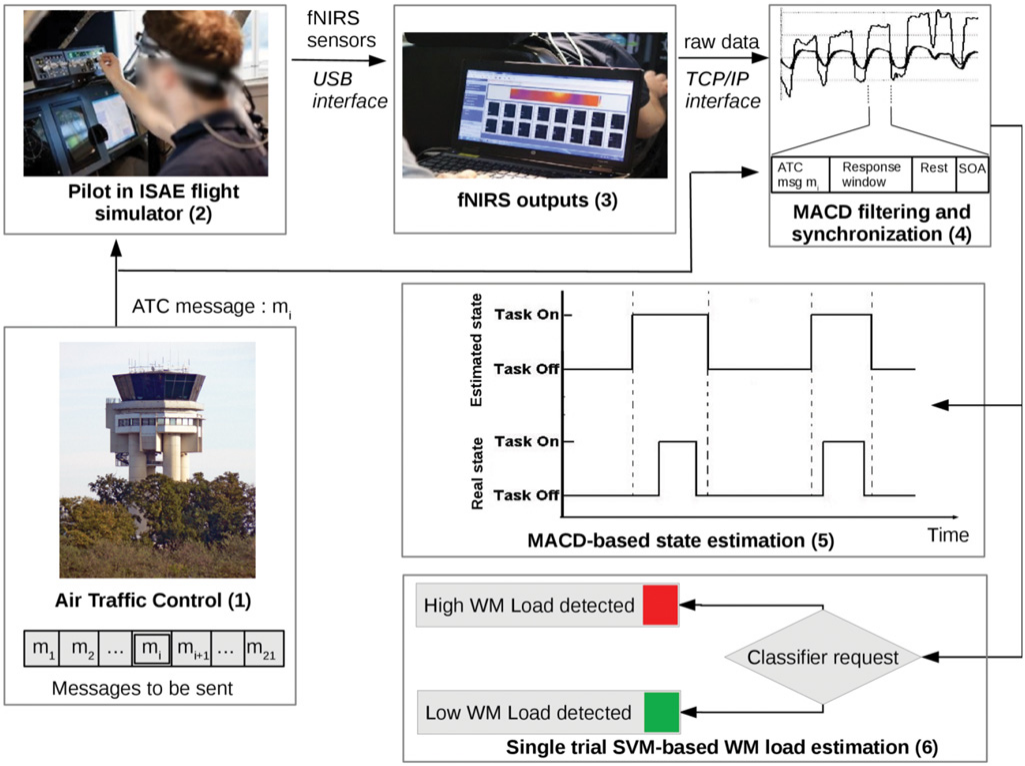
Illustration of the fNIRS based inference system. Pre-recorded ATC messages were sent to the pilot (1). The pilot’s prefrontal activity was measured with a fNIRS device (2). Output measures (3) were MACD-filtered and synchronized with the temporal design of the trial (4). During the entire session, the MACD-based state estimator detected whether the pilot’s state was *not-on-task* or *on-task* (5). When all of the required data were available for the trial, a request was sent to the pilot’s classifier to assess the WM load of the trial (6).

We implemented a WM load estimator that integrated different components (Fig. 4):

- a simulated ATC which broadcasts a list of chosen messages to the pilot;
- the ISAE flight simulator which allows a pilot to be in an ecological flight condition (cf. section 2.2);
- a fNIR100 sensor which measures the prefrontal oxygenation on 16 channels (cf. section 2.1);
- a MACD filter for artifact removal (cf. section 4.1);
- a synchronization module that also formats filtered data for the classification process: filtered fNIRS output must be synchronized with the pilot’s state, according to the instant of the arrival of that incoming message and according to the pilot’s response window;
- a state estimator (cf. section 4.2) which evaluates pilot’s instantaneous current state in real-time, which can be *not-on-task* or *on-task*. The pilot is considered *on-task* during ATC message reception, and *not-on-task* in the other periods.
- a classifier (cf. section 4.3) which evaluates in real-time whether the last ATC instruction was a *high WM load* trial or a *low WM load* trial.

Task monitoring, data acquisition and computation were conducted on the same computer (core i5-3210M, 2.50GHz, 4GB RAM).

#### 3.3 Experimental Time Course

**Fig 5:**
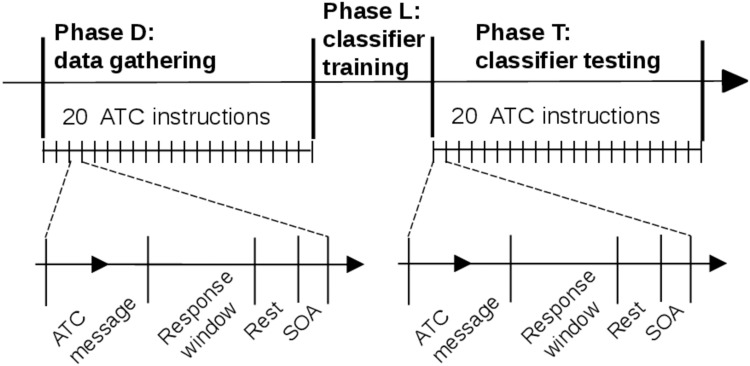
The experiment was split into three successive phases. Data gathering (*phase D*) and classifier testing (*phase T*) consisted of 20 ATC instructions each. The pilot’s classifier was trained between these two phases (*phase L*). The time scale of the figure is illustrative.

For machine learning purposes (cf. section 4.3), the experiment was split into three successive phases (Fig. 5):

- Phase D—data gathering phase: 20 instructions with two levels of difficulty were successively presented to the pilot in a random order. During phase D, the correctness of the pilot’s response was also checked for further pilot performance analysis. Entered FCU parameters were available through the ISAE flight simulator software. The fNIRS’s data were processed and recorded for each trial’s response window. The levels of difficulty of the message were also recorded.
- Phase L—classifier training phase: the classifier training process was activated, based on the data gathered during phase D. This phase was not perceived by the pilot (cf. section 3.2) and allowed further classification actions. At the end of this phase, the pilot’s classifier—the pilot’s specific classification model, correctly trained—was available for classification requests.
- Phase T—classifier testing phase: 20 instructions with random levels of difficulty (*high WM load* or *low WM load*) were successively presented. The aim of the classification process was to discriminate the difficulty of the trial, as soon as possible (cf. section 3.2). After each response window of trials, the classifier returned WM load estimation of the trial.

The transition from phase D to phase T was transparent to the participants.

### 4 Data Analysis

#### 4.1 MACD Filter

Raw fNIRS data were real-time filtered using a MACD filter, commonly used in economic market analysis [55]. This filter, based on the difference between a short-term EMA and a long-term EMA, implements a second order band-pass filtering to eliminate low-frequency (< 0.02Hz) and high-frequency (> 0.33Hz) components from the raw fNIRS signal [48]. This low order filter has a quasi linear phase in its bandwith and is particularly suited for real-time applications. For the experiment, we proceeded to an on-line filtering of Δ[*HbO*_2_] and Δ[*hHb*] on 14 of the 16 channels (Channels 8 and 10 were excluded due to several artifact acquisition issues), as described in Equation 1, where *N* represents the number of time points defining the EMA window:

$$\begin{array}{c} y=EMA_{N}\left(x\right)\Leftrightarrow y_{n}=\frac{2}{N+1}x_{n}+\frac{N-1}{N+1}y_{n-1}\\ MACD_{N_{short},N_{long}}\left(x\right)=EMA_{N_{short}}\left(x\right)-EMA_{N_{long}}\left(x\right) \end{array}$$ (1)

We chose a 6 s short-term EMA (*N*_*short*_ = 12) and a 13 s long-term EMA (*N*_*long*_ = 26), according to previous work [50] for MACD filtering, to get the desired bandwidth.

#### 4.2 MACD-based State Estimation

We performed MACD analysis to estimate the participant’s instantaneous mental state, *on-task* versus *not-on-task*, in real-time. In economic market analysis, Appel [55] states that a sustainable increase in the signal can be predicted when the MACD line crosses the signal from below. On the contrary, a sustainable decrease in the signal can be predicted when the MACD line crosses the signal line from above. This method can help estimate task-onsets and task-offsets based on the fNIRS signal [50]. We computed in real-time a state estimation chronogram by associating the moments when the MACD line crossed the signal line from below with stimuli onsets. To do this, we averaged MACD-filtered fNIRS data over the 14 channels. A *signal line* was computed using a 5 *s* EMA (*N* = 10) of this data, as described in Equation 2:

$$Signal\left(x\right)=EMA_{10}\left(MACD\left(x\right)\right)$$ (2)

**Fig 6:**
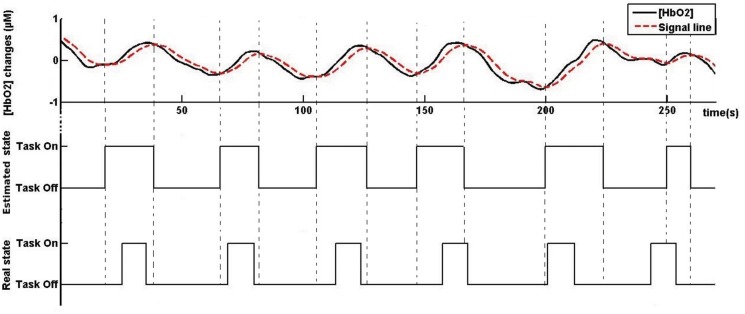
Example of real-time state estimation (performed on pilot 16). The upper graph shows MACD-filtered fNIRS signal and the signal line computed from the latter (dashed line). The two lower graphs show the participant’s state estimated from crossovers between MACD and signal lines and the operator’s actual state, respectively.

Similarly, we defined task-offsets when the MACD line crossed the signal line from above (for an example, see Fig. 6). We then compared each time point of the state estimation chronogram to the actual task-onsets chronogram in order to estimate the accuracy of this method. We labeled the state estimation at time *t* as:

- *Correct Estimation*, if the estimated state and the actual task state matched;
- *False Positive*, if the state was estimated as *on-task*, and actual state was *not-on-task*;
- *False Negative*, if the state was estimated as *not-on-task*, and actual state was *on-task*;

#### 4.3 Single Trial SVM-based WM Load Estimation

The classification’s goal was to discriminate on-line whether the last trial was a *high WM load* trial or a *low WM load* trial. For each pilot, we used the first 20 trials to train the pilot’s classifier (phase D and L, see section 3.3). From trial 21 to 40, we used the pilot’s classifier to discriminate trial difficulty, without any further training. An accuracy score of the pilot’s classifier was provided at the end of the experimental session.

Δ[*HbO*_2_] and Δ[*hHb*] signals were segmented into trials, in real-time, according to the task synchronization module (cf. section 3.2). Trial data were filtered and all the features described below were computed as soon as the fNIRS data were available.

We used a sliding-window on the trial data set to take into account the pilot’s personal hemodynamic response characteristics [52, 53]. A sliding-window was defined by:

- an offset (first time value of the sliding-window);
- a time interval (length of the sliding-window).

Fourteen prefrontal areas (all channels, except 8 and 10) corresponding to the channels with sufficient signal reliability were monitored (in terms of *HbO*_2_ and *hHb* concentrations, relative to a baseline) with our fNIRS system (cf. section 2.1).

From the Δ[*HbO*_2_] and Δ[*hHb*] signals, four time-domain features were calculated. For the sake of simplicity, Δ[*HbO*_2_]_*t*_ and Δ[*hHb*]_*t*_ correspond to relative concentration of *HbO*_2_ and *hHb* respectively during trial *t*, filtered with the MACD filter (cf. section 4.1), averaged during a specified time period.

We defined ${\bar{A}}_{\Delta {\left[HbO_{2}\right]}_{t}}$ (resp. ${\bar{A}}_{\Delta {\left[hHb\right]}_{t}}$), the mean amplitude response of the considered sliding-window of Δ[*HbO*_2_]_*t*_ (resp. Δ[*hHb*]_*t*_), on trial *t*, as the mean value within a specified time window of the trial, reported to a 2 s average pre-trial onset value, to take into account potential drift on fNIRS signal.

We used the following as features:

- mean Δ[*HbO*_2_]_*t*_ and mean Δ[*hHb*]_*t*_ on each voxel during the sliding-window of trial *t*;
- ${\bar{A}}_{\Delta {\left[HbO_{2}\right]}_{t}}$ and ${\bar{A}}_{\Delta {\left[hHb\right]}_{t}}$ on each voxel during the sliding-window of trial *t*;
- Δ[*HbO*_2_]_*t*_ and Δ[*hHb*]_*t*_ kurtosis (“peakedness” of the probability distribution) on each voxel during the sliding-window of trial *t*;
- Δ[*HbO*_2_]_*t*_ and Δ[*hHb*]_*t*_ skewness (asymmetry of the probability distribution) on each voxel during the sliding-window of trial *t*;

Sliding-window parameters were chosen according to previous findings concerning expected hemodynamic response [52, 53] and best classifiers features characteristics defined by Tail *et. al* [54]. Therefore, we defined sliding-window length according to three values (5s, 10s, 15s), and to seven different offsets (10s, 11s, 12s, 13s, 14s, 15s, 16s). These variations provided 168 predictors (3×7 sliding-windows, 8 features) for each channel. Then, on each trial, 2352 predictors were provided. Two classes had to be discriminated, *high WM load* trials and *low WM load* trials. As our number of features was large compared to the training sample, we used a linear Support Vector Machine (SVM) [60]. The principle of the SVM is to find the separating hyperplane that maximizes the distance between the hyperplane and the closest training points in each class. To avoid over-fitting, we chose to customize the SVM regularization parameter for each pilot’s classifier. In a linear SVM, the regularization parameter C controls the trade-off between errors of the SVM on training data and margin maximization. During the training process of each participant, the parameter C is incrementally changed over a large range of values (from 10^−5^ to 10). For each value, a cross validation step was performed and the parameter with the highest performance was chosen. The classifier was trained using a cross-validation (5-fold, 10-time) on the first 20 trials with the caret R packages [61]. The classifier training (phase L) was performed as soon as the data of the first 20 trials were available.

#### 4.4 Behavioral and Offline fNIRS Data

We performed classical off-line behavioral analysis to ensure that we correctly manipulated WM load (*i.e.* increased error rates in high WM load condition) and that the participants’ performance was identical across the two blocks (*i.e.* identical error rate during the first 20 trials and the last 20 trials). A two-way Analysis of Variance (ANOVA) was carried out on the correct response rate between subject factors Phase (Learning vs. Test) and Position (Speed vs. Heading vs. Altitude vs. Vertical speed). An off-line analysis on the neurophysiological data to verify the consistency of prefrontal activation with existing neuroimaging literature was performed. To do so, we computed the frontal [*HbO*_2_] and [*hHb*] peak response (peak value within 30s post-trial onset minus 2s average pre-trial onset) for each trial and each pilot using the MACD-filtered data. We then performed a three-way ANOVA using within subject factors Oxygenation (*HbO*_2_ vs. *hHb*), WM Load (High vs. Low) and Voxels (1 vs. 2… vs. 16), excluding voxels 8 and 10 due to several acquisition artifact issues. Tukey’s HSD post-hoc tests were used to evaluate all behavioral and hemodynamic interaction effects.

## Results

### 1 Behavioral and Physiological Results

The participants committed a mean of 13.2 errors (*SD* = 4.7) during the entire experiment, all occurring during the high load trials. All the subjects completed the low WM load trials correctly. There was no significant effect of the phase (learning or testing) on the number of committed errors.

**Fig 7:**
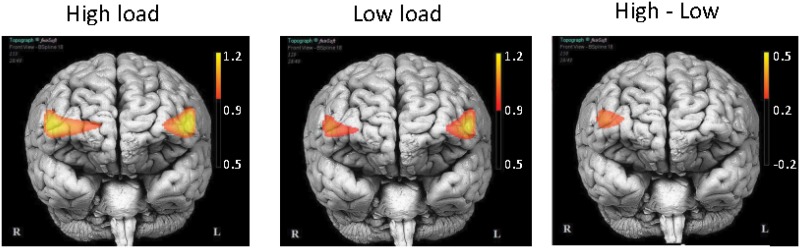
Activation maps according to the level of difficulty. Units are in *μmol*.*l* ^−1^. Both high and low load conditions elicit bilateral DLPFC activities. The high load minus low load subtraction map (High—Low) shows significantly greater activation of the right DLPFC. Activations shown 14 s post-stimulus onset. *p* < 0.001. fNIRSOFT® software (www.biopac.com/fNIR-Software-Professional-Edition) was used to produce this figure.

The ANOVA over the fNIRS data revealed a main effect of the oxygenation (*F*(1, 18) = 95.2; *p* < 0.001; partial *η* ^2^ = 0.90) with higher [*HbO*_2_] than [*hHb*] and a main effect of the load (*F*(1, 18) = 7.3; *p* < 0.05; partial *η* ^2^ = 0.29) corresponding to higher peak response within the high load condition. In addition, a significant interaction effect between load and oxygenation was found (*F*(1, 18) = 28.7; *p* < 0.001; partial *η* ^2^ = 0.61) showing that the load effect was only present for [*HbO*_2_] (*p* < 0.001). Finally, a second order interaction effect revealed that the load effect was not homogeneous across voxels (*F*(13, 221) = 2.87; *p* < 0.001; partial *η* ^2^ = 0.14). Post-hoc tests revealed a maximum load effect within the right DLPFC (i.e, voxel 15; see Fig. 7 for illustration) for[*HbO*_2_].

### 2 MACD-based State Estimation Results

#### 2.1 Accuracy

The real-time estimated state was compared to the actual state of the subject during the experiment (*not-on-task* or *on-task*). The results show that the estimation matched 61.74% of the time (*SD* = 4.27%), which was significantly better than chance (*F*(1, 18) = 145.52; *p* < 0.01) (Fig. 6). We obtained a 58.24% mean specificity (SD = 3.80%), and a 71.88% mean sensitivity(SD = 9.34%).

#### 2.2 Latencies

The required time for data filtering (maximum per sample < 0.4 *ms*) is negligible regarding fNIRS time resolution (2Hz). Hence, pilot’s state estimation (*not-on-task* or *on-task*) is available in real-time.

#### 2.3 Off-line Analysis

**Fig 8:**
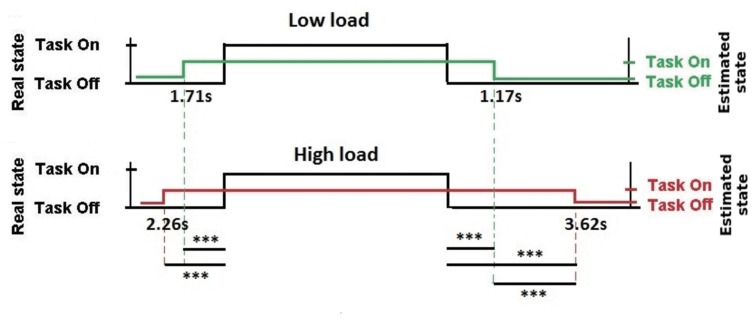
Off-line estimated onset and offset latencies compared to the stimuli onset, in low WM load and high WM load conditions. Average for 20 trials per difficulty, on 19 pilots’ results. ***: p<0.001.

The results of estimated onsets and offsets latencies compared to stimuli onsets and offsets are summarized in Fig. 8. On average, the onset of state estimation significantly occurred 1.97*s* before the actual state onset (*SE* = 0.34*s*). The estimated offset occurred 2.43*s* after the stimulus offset (*SE* = 0.52*s*).

The difficulty of the current trial had no effect on the onset estimation latency (*p* = 0.15). Concerning the offset estimation latency, the offset estimations occurred significantly later for high load trials (*Mean* = 3.62*s*; *SE* = 0.66*s*) than for low WM load ones (*Mean* = 1.17*s*; *SE* = 0.59*s*) (*F*(1, 18) = 12.3; *p* < 0.01).

### 3 Single trial SVM-based WM Load Estimation Results

#### 3.1 Accuracy

**Fig 9:**
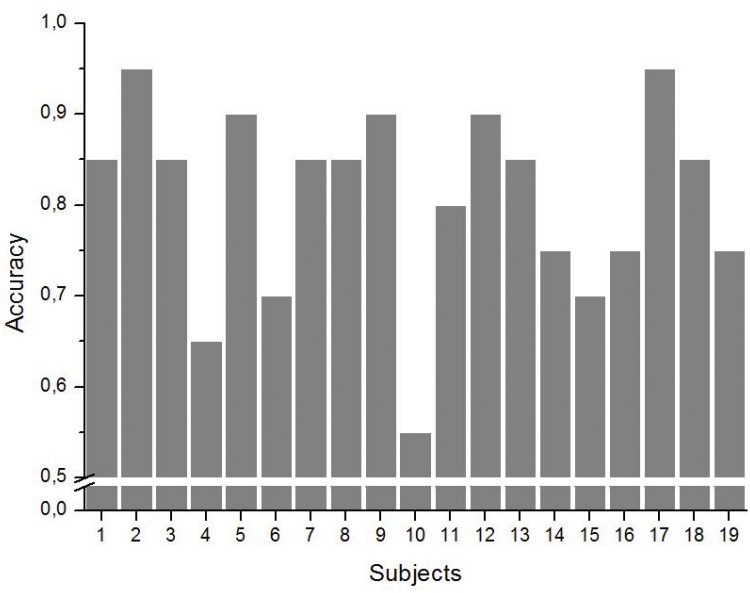
Machine learning result: WM Load level estimation accuracy for each participant.

During the testing phase, a mean of 80.8% (*SD* = 10.6%) of the trials were accurately classified (discriminated into on-line *low WM load* trials and *high WM load* trials). We obtained a 72.11% mean specificity (*SD* = 19.89%), and a 89.47% mean sensitivity (*SD* = 15.72%). Individual classifiers’ accuracies are shown in Fig. 9.

#### 3.2 Latencies

After phase D was completed, the classifier training script was run concurrently with the 21^*st*^ trial achievement. This process took a maximum of 42.15*s*. In the worst case, it can delay the 21^*st*^ trial WM load estimation result.

**Fig 10:**
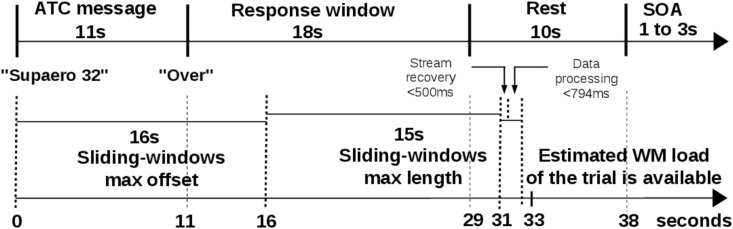
Trial timeline and computing latencies. The upper timeline shows ATC span task trial events duration (see Fig. 3). Bottom timeline illustrates duration constraints to get pilot’s estimated WM load: classifier’s response is available in the worst case less than 3.3*s* after pilot’s response window.

During classifier’s training (phase T), a request to the classifier and its associated response took a maximum of 770*ms*. This request can be sent as soon as the trial’s filtered data set is available. In fact, due to the machine learning design (cf. section 4.3), all the trial data set required for a request to the pilot’s classifier is constrained by the maximum sliding-window offset (16*s*) and the maximum sliding-window length of 15*s* (Fig. 10). Therefore, the trial data set is theoretically available 2*s* after the pilot’s response window. The required time for data filtering and formatting is negligible regarding fNIRS time resolution (2Hz), even for a full trial data set (maximum < 24 *ms* of computing time on a complet trial data set). However, the classifier’s process takes 770*ms* in the worst case to return an estimated WM load for the trial. The pilot’s estimated WM load is then available less than 3.3*s* after his response windows.

## Discussion

The objective of this study was to implement on-line tools to infer pilots’ cognitive activity [62]. We focused on the monitoring of WM as this executive function is highly solicited when operating aircraft [1, 2]. The design of such an inference system was challenging as, until now, only three studies involving on-line fNIRS-based state inference systems in an ecological context [39–41] have been conducted. However, these studies did not include realistic simulators but simplified PC-based simulations. In order to test our inference system, an experimental protocol was designed, during which the pilots had to interact with ATC instructions of two levels of difficulty. The behavioral results confirmed that these levels were contrasted, as participants performed less well during the higher difficulty level. This result is coherent with Taylor *et al.* [9] which has shown that pilots’ WM decline when four different instructions have to be stored and recalled. The neurophysiological results also confirmed that the task difficulty statistically modulated oxygenation level in the prefrontal cortex (PFC). Moreover, the topographic maps (Fig. 7) revealed particular activations of the right and left dorsolateral PFC (DLPFC; BA 9 and 46) that are seen as mediating monitoring, i.e., executive control in the Baddeley’s model of WM [63]. Indeed, these results, in accordance with previous findings [20–24], confirmed that fNIRS is a suitable device for monitoring WM load level.

### 1 MACD-based State Estimation

One novelty of this study was the use of a MACD filter as a systematic state estimator to detect the pilot’s state (doing or not doing a WM task) [50]. The results were promising as the MACD-based state estimation matched 62% of time with the real duration of *on-task* and *not-on-task* activity, i.e. receiving (memorizing) or not the ATC instruction (Fig. 6), with a good true positive rate (72%). The differences observed were explainable by the presence of 42% false positive rate, when the pilot’s state was estimated as *on-task* and the stimulus’s state was *not-on-task*. Our off-line analysis revealed that, on average, the *on-task* estimated periods started before and ended after the stimulation periods (Fig. 8). Furthermore, the delay between stimulus onset and state estimation offset was significantly higher during the high WM load trials than during the low WM load ones, suggesting a potential different way of dissociating workload levels. According to our definitions of the real and the estimated states, it is of a great importance to consider that the estimated pilot’s WM load could not perfectly fit to the real state as ATC messages are physical stimuli and pilot’s state is the ATC brain-related activity that necessarly extends beyond physical stimuli. To this extent, the latencies observed could be imputable to the anticipation of stimuli onset due to task rhythmicity and the maintenance in WM of ATC instructions, two functions that have neural substrates in the PFC [64]. Altogether, these results are consistent with previous studies proving the potential of fNIRS for idle mode detection [26, 47]. In fact, fNIRS offers an insight into the brain’s reaction to stimuli, giving information on perceived workload that would not be available through behavioral measures (e.g. stimulation periods). The results also confirmed that MACD is an effective method for real-time task onsets detection [50], requiring no *a priori* information on task onsets, few computational resources, and no calibration. This result is key for ecological tasks, when onsets timing are unknown. For example, in realistic situations when events happen randomly, the information concerning task onsets could be retrieved using such a system. This method would provide a simple and systematic way to trigger classification algorithms for workload level assessment, in addition to a *not-on-task*/*on-task* mode detection.

### 2 Single Trial SVM-based WMLoad Estimation

Along with the state estimation, we used machine learning techniques to discriminate *low WM load* versus *high WM load* trials. The mean classification accuracy reached up to 80.8% successful discrimination between low and high WM load trials with good mean specificity (89.5%) and sensitivity (72.1%). Moreover, the mean accuracy for 16 out of 19 participants was equal or superior to 70%, defined as a sufficient rate for BCI [54, 65]. Only one participant’s WM load level estimation had a lower-than-chance classification. In fact, these results compare well to the rare on-line studies such as the ones conducted by Naseer *et al.* [38] (14 participants: 82.14% accuracy), Girouard *et al.* [39] (9 participants: 83.5% accuracy), and Schudlo *et al.* [43] (10 participants: 77.4% accuracy). However one has to consider that two of these studies involved controlled “rest versus task” paradigm [38, 39], a two-class problem that leads to better classification results than a “low WM load versus high WM load” one. Indeed, these results demonstrated the efficiency of considering both spatial and temporal features as proposed by Tai *et. al* and Schudlo *et. al* [43, 54], that allowed the monitoring of WM load level in ecological situations.

Another key issue when assessing the performance of an on-line inference system is related to the delay of single trial classification. Here, the automatic classification of WM load level occurred in the worst case less than 21.3*s* after the end of the ATC instruction (ATC instructions last 11*s* and estimated WM load of the trial is available less than 32.3*s* from the beginning of the trial), a comparable result with other on-line fNIRS-based BCI latency (for a review of on-line fNIRS-based BCI latency, please refer to Strait *et. al* [35]). Such a delayed inference was not an issue in our experimental situation as pilots had 18*s* to program the autopilot according to the ATC clearances. As a matter of fact, the diagnosis of the WM performance (*high WM load* or *low WM load*) occurred at maximum 3.3*s* after the end of the participants’ task (Fig. 10). Although the loop was not completely closed in our study, this signal could be used to automatically give a feedback, for example to ATC. Such a feedback could allow ATC to check that the instruction has been understood, or to repeat the instruction to the pilots when high WM load conditions are detected, with an acceptable 3.3*s* maximum latency.

### 3 Limitation of this Study and Perspective

These results offer promising perspectives towards the design of a fNIRS-based “passive” BCI for pilots. However, its use in real operational cockpit still remains a challenge as safety is critical in aeronautics.

First, we used a simplified pilot/ATC task for an easy implementation of our inference system. Since ATC was simulated, it prevented mutual pilot—controller verification as in a real operational situation. Despite this ecological limitation, we believe that our approach was relevant and could address several issues in aviation such as pilots training, pilot selection, or the monitoring of pilots’ WM ability that is highly sollicited when interacting with ATC [18]. In the future, pilots will use data-link technology that presents ATC messages as text to limit pilot-ATC communications. Data-link changes the nature of pilot-ATC interactions and several studies have shown that it may negatively impact pilots’ WM performance especially when data-link interferes with other concurrent tasks [6, 8]. Our next challenge is to test our real-time inference system in a multitasking context (i.e. ATC messages and failures management) and to dynamicaly adapt the interaction depending on pilots’ spare capacity.

A second issue in the use of a BCI in aviation is related to its reliability. A lack of BCI reliability could trigger spurious assistance and thus impair global pilots’ performance. Indeed, a mean classification rate of 80.8% and a 3.3*s* delayed diagnosis of WM load level cannot guarantee that the interaction is adapted in a timely and accurate manner. Therefore, a first step to refine this approach is to integrate complementary measurements such as EEG [66, 67] or physiological sensors [68] that have been shown to significantly enhance classification performance when combined with fNIRS. A second step is to benchmark other machine learning techniques such as Hidden Markov Models [69] or neuro-fuzzy inference systems [70] that are well suited for the processing of physiological data [71]. Furthermore, once our classifier was trained (*i.e.* phase L), it remained static. Reinforcement learning [72] should be considered for gathering more samples and for updating our classifier with on-going trials. Such an approach would allow more robustness with potential optodes position drifting issues across long sessions and would permit to take into account participants’ neurophysiological evolutions across time (e.g. fatigue, circadian rhythm [73]). Another way of improvement could be to use more than one classifier method at the same time [74, 75], as shown in practice by Tai *et. al* [54]. Moreover, this approach would allow for better classification of naturalistic ATC stimuli that are not at the extremes of a high vs a low WM load continuum. Another perspective is to explore techniques to speed up response detection on fNIRS signal such as the ones proposed by Cui *et. al* [49] that can drastically reduce latency in detecting change in a mental state.

Finally, lingering issues remain regarding the implementation of a BCI in the cockpit. Aircraft accelerations (i.e. “G-forces”) may impact blood flow or creates headband motion artifacts [76]. Controlled experiments have to be conducted in real flight with the use of accelerometers to assess their effects on blood flow before the implementation of such systems in operational conditions. The usability of the BCI is another key factor in the acceptance of this technology. No pilot would accept a lengthy calibration process to train the classifier before each flight departure. From a scientific point of view, this problem addresses inter-session consistency that is the analysis of the consistency of spatial and temporal features of cerebral oxygenation while performing a similar task across time. Some authors have investigated promising tracks and have shown that it is possible to define a trade-off between accuracy and calibration time [77]. The re-use of data collected during previous sessions [78] and the identification of potential users profile could help in dealing with this critical issue.

## Supporting Information

S1 Dataset Raw data from COBI Studio® used in this study. (BZ2)
